# Molecular interaction and cellular studies on combination photodynamic therapy with rutoside for melanoma A375 cancer cells: an in vitro study

**DOI:** 10.1186/s12935-020-01616-x

**Published:** 2020-10-29

**Authors:** Khatereh Khorsandi, Reza Hosseinzadeh, Elham Chamani

**Affiliations:** 1 Department of Photodynamic, Medical Laser Research Center, Yara Institute, ACECR, Tehran, Iran; 2 Department of Medical Laser, Medical Laser Research Center, Yara Institute, ACECR, Tehran, Iran; 3 Cardiovascular Diseases Research Center, Birjand University of Medical Sciences, Birjand, Iran; 4 Department of Clinical Biochemistry, Birjand University of Medical Sciences, Birjand, Iran

**Keywords:** Photodynamic therapy, Melanoma, Rutoside, Cell cycle, ROS, Apoptosis

## Abstract

**Background:**

Melanoma as a type of skin cancer, is associated with a high mortality rate. Therefore, early diagnosis and efficient surgical treatment of this disease is very important. Photodynamic therapy (PDT) involves the activation of a photosensitizer by light at specific wavelength that interacts with oxygen and creates singlet oxygen molecules or reactive oxygen species (ROS), which can lead to tumor cell death. Furthermore, one of the main approches in the prevention and treatment of various cancers is plant compounds application. Phenolic compounds are essential class of natural antioxidants, which play crucial biological roles such as anticancer effects. It was previously suggested that flavonoid such as rutoside could acts as pro-oxidant or antioxidant. Hence, in this study, we aimed to investigate the effect of rutoside on the combination therapy with methylene blue (MB) assisted by photodynamic treatment (PDT) using red light source (660 nm; power density: 30 mW/cm^2^) on A375 human melanoma cancer cells.

**Methods:**

For this purpose, the A375 human melanoma cancer cell lines were treated by MB-PDT and rutoside. Clonogenic cell survival, MTT assay, and cell death mechanisms were also determined after performing the treatment. Subsequently, after the rutoside treatment and photodynamic therapy (PDT), cell cycle and intracellular reactive oxygen species (ROS) generation were measured.

**Results:**

The obtained results showed that, MB-PDT and rutoside had better cytotoxic and antiprolifrative effects on A375 melanoma cancer cells compared to each free drug, whereas the cytotoxic effect on HDF human dermal fibroblast cell was not significant. MB-PDT and rutoside combination induced apoptosis and cell cycle arrest in the human melanoma cancer cell line. Intracellular ROS increased in A375 cancer cell line after the treatment with MB-PDT and rutoside.

**Conclusion:**

The results suggest that, MB-PDT and rutoside could be considered as novel approaches as the combination treatment of melanoma cancer.
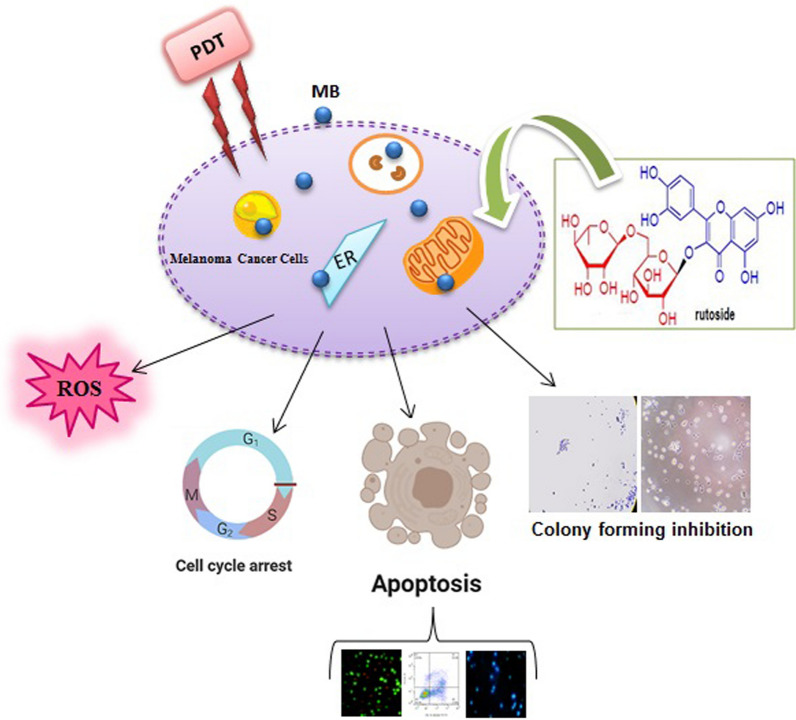

## Background

Melanoma is originating from malignant changes in the melanocyte cells, which can produce the epidermal pigment. Melanoma is invasive and malignant form of skin cancer. Almost 75% of deaths from skin malignancies are caused by melanoma [1, 2]. About 100,350 new cases are reported in 2020 in both genders in US, which include 60,190 male cases and 40,160 female cases. Moreover, about 6850 estimated death cases in both genders were reported, including 4610 male cases and 2240 female cases [3]. Melanoma could be treated if diagnosed at early stages; however, approximately 20% of the advanced melanomas are diagnosed as being resistant to treatment. This case emphasized the role of early diagnosis, just as an advancement in the treatment of melanoma [4–6].

There are different strategies for the treatment of melanoma cancer such as chemotherapy, radiotherapy, and immunotherapy, which most of them have some side effects and consequently lead to cancer cell resistance. Up to now, many studies have suggested various alternative therapies for increasing therapeutic efficacy of melanoma drugs and reducing their resistance using the combination therapy [7], low level laser irradiation [8], reducing cholesterol [9], and controlling obesity [10, 11].

Photodynamic therapy (PDT) is a newly technique for the treatment of different diseases, which can kill the damaged cells (such as cancer cells or cells infected with microorganisms, etc.) or unwanted tissues (for example; removeing the atherosclerotic plaques in the arteries). The photodynamic therapy basis contains the stimulation of a non-toxic compound called photosensitizer (PS) by specific light to generate reactive oxygen species (ROS) that can kill the cells [12, 13]. An ideal PS has several features such as non-toxicity, selective removal and maintenance by tumor tissue, and production of free oxygen radicals by absorbing wavelengths that can easily pass the tissue [14, 15]. For performing a safe and effective PDT, an photosensitizer at therapeutic concentrations should be sent to the target cell (tumor cell), which would be then absorbed in small quantities by non-target cells, thereby minimizing the unintended side effects in healthy tissues [16]. One of the fundamental elements in enhancing the PDT efficiency is the selection of appropriate PS. Many light-sensitive materials have been characterized by different physico-chemical properties, each with its own advantages [17–19]. Methylene blue (MB), as a PS, was used in the present study, which is a blue cationic phenothiazine that becomes colorless when reduced. Methylene blue's advantages over other light sensors include its ability to bind with mitochondria, induce apoptosis, produce free radicals under hypoxic conditions, not be repelled by drug resistant cancer cells, and capability of being activated by various light sources [20, 21]. Various experiments have demonstrated the use of MB in the successful photodynamic treatment of some tumors [20, 22–30]. Bioactive components from the plants have been confirmed for their anti-cancer activities, which play main roles in the discovery and the development of various drugs [31–33]. Nowadays, scientists refer to flavonoidsas a special group of therapeutic molecules [34]. Accordingly, one of these important flavonoid compounds called rutoside was found in some plants such as mud, buckwheat, tea leaves, and apples. The word rutoside is derived from the plant name *Ruta graveolens*, which is rich in rutoside. In addition, rutoside is also known by other names such as vitamin p, quercetin-3-*O*-rutinoside and sophorin [35]. Furthermore, Rutoside has many medicinal and therapeutic properties such as antioxidant activities, anti-cancer [36–38], anti-diabetic, nervous system protection, and antibacterial effects [39–41]. In this regard, the purpose of this study was to combine the photodynamic therapy with rutoside substance to find the effect of rutoside of MB-PDT efficiency. Herein, the rutoside interaction with MB and their role in PDT treatment on A375 melanoma cells were studied.

## Materials and methods

### Materials

Rutoside, 3-(4,5-dimethylthiazol-2-yl)2,5-diphenyltetrazoliumbromide (MTT), Tryphan blue solution 0.4%, Acridine orange, ethidium bromide,Hoechst, and dimethyl sulfoxide (DMSO) were obtained from Sigma-Aldrich (St Louis, MO, USA). Fetal bovine serum (FBS), phosphate buffer saline (PBS), and antibiotics were purchased from Gibco (Gibco BRL). Dulbecco’s Modified Eagle Medium (DMEM) was obtained from Invitrogen (Invitrogen, Carlsbad, California, US). All the other reagents were bought from Merck.

### Methods

#### Spectrophotometric study of methylene interactions with rutoside

Photosensitizer [methylene blue (MB)] (10 µg/mL) and rutoside stock (1 mg/mL) were prepared by dissolving a particular range of each in double distilled water. Alternations of methylene blue UV/Vis spectrum with the enhanced concentrations of rutoside were recorded at 500–800 nm wavelength by the use of water as a blank. The obtained information were analyzed, and molecular binding constants were determined by employing an appropriate theoretical procedure.

#### Cell culture

Melanoma cancer cell (A375) and human dermal fibroblast cell lines (HDF) were obtained from the Institute of Pasture, Tehran, Iran. These cells were negative for mycoplasma, bacteria, and fungi andgrown in DMEM medium that was supplemented with 10% FBS, 100 IU/mL penicillin, and 100 μg/mL of streptomycin, which were then incubated in a humidified incubator containing 5% CO_2_ at 37 °C. For performing further experiments, the cells were removed by trypsinizing (trypsin 0.025%, EDTA 0.02%) and then washed with PBS. Ethical approval was obtained from the Research Ethics Committee of Birjand University of Medical Sciences (IR.BUMS. Rec.1398.395).

#### Effect of different concentrations of rutoside on human cancer and normal cells

Briefly, the normal and cancerous cells (1 × 10^4^ cells) were seeded in 96-well plates using fresh DMEM culture medium, and then incubated under 5% CO_2_, for 24 h at 37 °C. Then, the cells were incubated using fresh cell culture medium containing different concentrations of rutoside (0, 5, 10, 25, 75, and 100 μg/mL). After the certain incubation time (4 and 24 h); the cells were washed by PBS solution. The MTT assay was then applied to measure the viability of the cells. Notably, each experiment was repeated 3 times, and the related data are represented as the mean ± SD.

#### In vitro photodynamic treatment

The normal and cancerous cells (1 × 10^4^) were incubated for 1 h with different concentrations of methylene blue (MB) (0, 1, 5, 10,15, and 25 μg/mL). Thereafter, the cells were washed by PBS and irradiation was performed for 90 s using red light source (660 nm; power density: 30 mW/cm, 3 J/cm^2^). Subsequently, the MTT assay was applied to identify the viability of the cells. Notably, each experiment was repeated 3 times, and the obtained data are represented as the mean ± SD.

#### MTT assay

Thiazolyl blue tetrazolium bromide (MTT) was used for determining the cell viability. Cell viability can be measured as a function of the cell's redox potential. Living cells can change the MTT compound to an insoluble formazan. The resulting formazan can be solubilized by dimethyl sulfoxide (DMSO), and its concentration can be determined using spectrophotometric methods [42]. Briefly, the culture medium was removed, and the cells were incubated in medium containing 0.5 mg/mL of 3-(4,5-dimethylthiazol-2-yl)-2,5-diphenyltetrazolium bromide for 3–4 h at 37 °C. The obtained purple formazan crystals were dissolved in 100 μL DMSO, and then shacked for 15 min. The absorbance was measured at 570 nm using an ELISA reader (Hyperion, Inc., FL, USA). Each experiment was repeated 3 times, and data are represented as the mean ± SD.

#### Inverted light microscopy and colony-forming assay

To investigate the morphology changes of melanoma A375 cancer cells after the treatment with rutoside and MB-PDT, the cells were exposed to rutoside for 4 h and were then treated with MB for 1 h following irradiation (PDT). Afterward, the cells were studied using light inverting microscope at 40× magnification. For performing the colony assay, the treated cells were collected and total numbers of cells were counted, and then 200 cells/plate were seeded. Following one-week incubation at 37 °C, colonies were stained with 0.5% crystal violet in methanol, and the number of colonies was counted. The control was the untreated cells that were kept for 24 h.

#### Apoptosis induction by rutoside and MB-PDT: AO/EB double staining, hoechst staining, and annexin V/PI flow cytometry analysis

For performing this experiment, the A375 cells (1 × 10^6^ cells) were separately seeded in the petri dish, and by passing 24 h from the incubation time in 5% CO_2_ at 37 °C, one petri was considered as the control (dark) and the other one was treated with rutoside (4 h), and then MB-PDT was performed as it described earlier. After 24 h, the cells were pelleted, resuspended in 100 µL of PBS, and were then stained with Acridine Orange/Ethidium Bromide (AO/EB) in terms of the published procedures [43]. The concentrations of AO (Sigma, USA- A6014) and EB (Sigma, USA-E7637) were considered to be 0.1 and 0.25 mM, respectively. The control and treated A375 cells were stained with Hoechst 33258 (1 mg/mL) and other steps were done as mentioned for AO/EB staining. Morphological alternation because of induction of apoptosis, were detected using fluorescence microscopy (BEL, Italy).

In order to determine the percentage of apoptotic cells in rutoside and then MB-PDT treated cells, and compare it with the control cell, the cancer cells were stained with Annexin-V and propidium iodide (PI) and were then incubated for 10 min at 25 °C in darkness. At the end, the cells were analyzed using flow cytometry. FlowJo 7.6.1 software was also used for data analyses.

#### ROS production in cancer cells

The intracellular ROS accumulation was measured using the 7.2-dichlorofluorosine diacetate (DCFH2-DA) assay [8]. For this purpose, A375 melanoma cancer cells were cultured in approximately 10^6^ cells per petri dish. Cells were treated with rutoside and then MB-PDT as described earlier. Cell culture was removed and the cells were incubated with 2 mM DCFH2-DA for 45 min in darkness. The cells were washed with PBS, and then transferred to a flow cytometer for performing ROS assay. The obtained data were analyzed using FlowJo 7.6.1 software.

#### Cell cycle analysis

Approximately 1 × 10^6^ A375 cells/cm^2^ were treated with rutoside and then MB-PDT. The cells were washed twice with PBS by centrifugation (200×*g*, 5 min, 4 °C) and were then fixed in cold 70% ethanol (24102; Sigma). The fixed cells in ethanol were kept at least 2 h at − 20 °C. Then, the cells were washed twice with cold PBS by centrifugation, and the cell pellet were resuspended in 300 μL of PBS containing 100 mg/mL RNAse (PR891628C; SinaClon BioScience, Tehran, Iran), 10 mg/mL PI (P4170; Sigma), and 10 mL of 0.1% (v/v) Triton X-100 (108643; Merck, Germany) for 15 min in darkness. The fluorescence emission of PI can be detected using excitation at 488 nm (blue) and emission at > 650 nm (red) wavelengths. Data were analyzed using Becton-Dickinson FACS Calibur Flow Cytometer and following by FlowJo 7.6.1 software.

#### Statistical analysis

Statistical analysis was performed using student’s t-test (two tailed). All values are expressed as means ± SD. *P* < 0.05 was considered as statistically significant.

## Results

### Interaction of methylene blue with rutoside

**Fig. 1:**
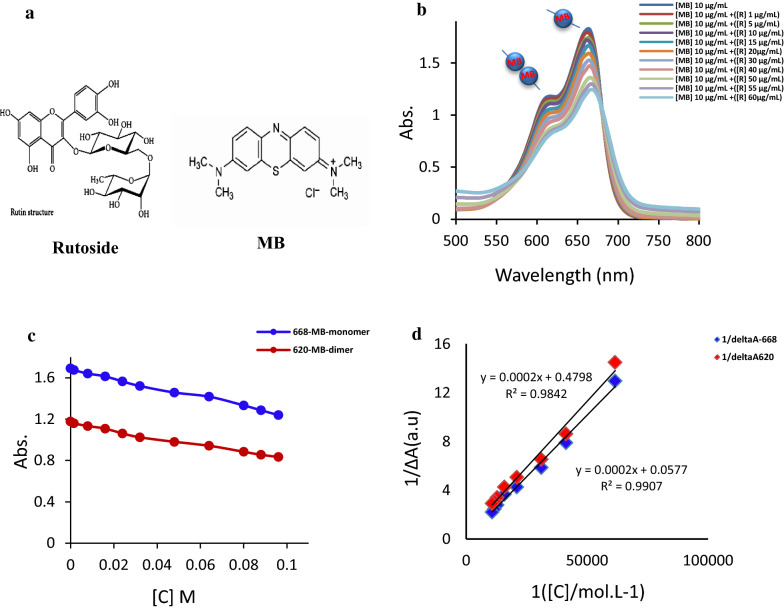
Schematic structure of rutoside (R) and methylene blue (MB) (**a**). Methylene blue UV/vis spectrum variations in the presence of different concentrations of rutoside (**b**). Alternation in absorbance at 620 nm (dimeric form of MB) (black circle) and 668 nm (monomeric form of MB) (black circle) by increasing rutoside (**c**). Benesi-Hildebrand plot for interaction of methylene blue (10 µg/mL) (dimeric form of MB) (red diamond suit) and 668 nm (monomeric form of MB) (blue diamond suit) with rutoside at pH = 7 and at 25 °C (**d**). *R* Rutoside, *MB* methylene blue

Spectrophotometric titration of methylene blue solution with rutoside (stock solution, 1.64 × 10^–3^ M) demonstrated the bathochromic shift in MB maximum absorption spectra (in both of dimer and monomer maximum absorption wavelengths) along with a reduction in absorbance by increase in the rutoside concentration. The results show that, rutoside can interacts with MB in monomeric and dimeric forms. Figure 1b shows the spectral change of MB solution by addition of enhanhing concentration of rutoside. In Fig. 1c, the variation of absorbance versus rutoside concentration was constructed based on the monomer and dimer methylene blue maximum absorption peaks. As shown in Fig. 1d, the variation of 1/∆Abs versus 1/[C] was constructed based on the Benesi Hidebrand equation [44]. The graph demonstrated that, the variations are linear and molecular interaction is 1:1 equilibrated in both of the dimeric and monomeric forms of methylene blue. The related binding constant can be estimated based on the well-known Benesi Hidebrand equation. By considering the binding constants, the Gibbs free energy of interactions can be achieved (Table 1) [45].

**Table 1:** Thermodynamic parameters related to the binding sets in MB interaction with rutoside, and obtained based on the Benesi–Hildebrand equation

	K_b_	Ln K_b_	∆G_b_/KJ/mol
Monomer MB-R	2399	7.782807	− 19.28
Dimer MB-R	288.5	5.664695	− 14.03

### Effect of rutoside on the normal and cancerous cells

**Fig. 2:**
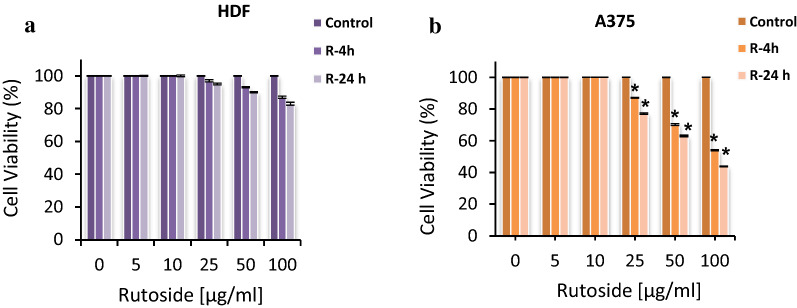
The cell viability of HDF fibroblast cells (**a**) and A375 melanoma cell (**b**) treated with different concentrations of rutoside for 4 and 24 h in darkness. The results are expressed as mean ± SD (n = 3), *P < 0.05 (compared with control (without rutoside/ only PBS) group. *R* rutoside

To investigate the cytotoxicity effect of rutoside in the absence of irradiation, the cell viability of the treated cells was determined with different concentrations of rutoside (0, 5, 10, 25, 50, and 100 μg/mL) after 4 and 24 h incubations. The cell viability of human dermal fibroblast cell line (HDF) in the presence of rutoside showed no significant change, and in higher concentration at 100 µg/mL after 4 h, it slightly changed to 83% (Fig. 2a). The results of the effect of rutoside on melanoma A375 cell line in the absence of light revealed that, the survival of melanoma cancer cells decreased in the presence of rutoside and the cell viability was 54% and 43% at the concentration of 100 μg/mL after 4 h and 24 h, respectively (Fig. 2b). According to the obtained results, the half maximal inhibitory concentration (IC_50_) for rutoside on melanoma A375 cell line after 4 and 24 h incubation was approximately 100 µg/mL. In the next step, we examined different strategies for the combination of rutoside and MB-PDT, as summarized in Table2.

**Table 2:** Different strategies for the combination of rutoside and MB-PDT

Different strategies	Pre-treatment/time	Treatment	Post-treatment/time	Cell lines
Pretreatment with rutoside for 4 h then MB-PDT	Rutoside—4 h	MB-PDT	–	A375 melanoma and HDF
Pretreatment with rutoside for 24 h then MB-PDT	Rutoside—24 h	MB-PDT	–	A375 melanoma
MB-PDT then post treatment with rutoside for 4 h	–	MB-PDT	Rutoside-4 h	A375 melanoma
MB-PDT then post treatment with rutoside for 24 h	–	MB-PDT	Rutoside-24 h	A375 melanoma
Simultaneous MB and rutoside for 1 h then PDT	–	Rutoside and MB for 1 h then PDT	–	A375 melanoma

### Pre-treatment effect of rutoside on MB-PDT toxicity

**Fig. 3:**
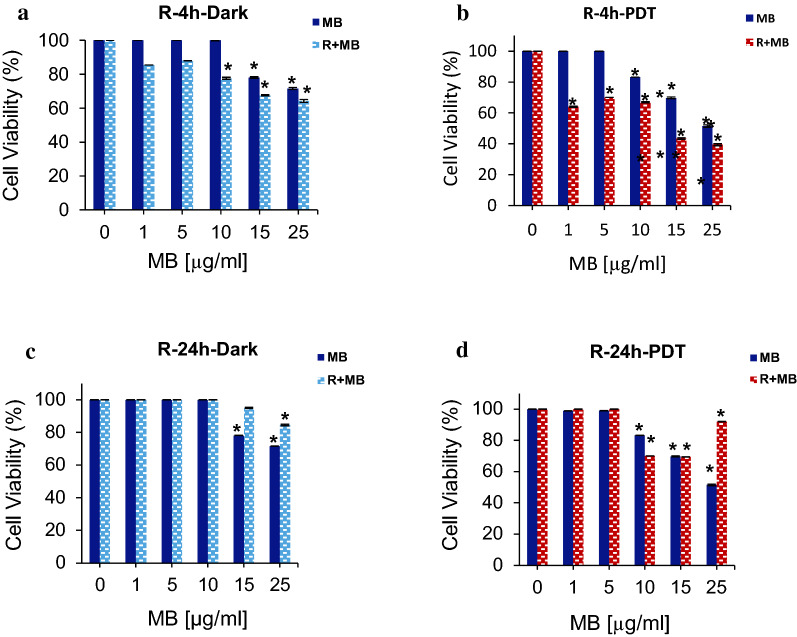
The cell viability of A375 melanoma cancer cells treated with various concentrations of MB and rutoside. Rutoside [50 µg/mL for 4 h (**a**, **b**) and 24 h (**c**, **d**)] and then MB treatment was performed for 1 h and keeping in darkness or with red irradiation (660 nm) for 90 s (PDT). The results are expressed as mean ± SD (n = 3), *P < 0.05 (compared to the control (untreated) group. *R* rutoside

To explore the effect of rutoside on MB-PDT, we designed series of experiments as follows: rutoside treatment as pre-treatment and post-treatment, and also rutoside and MB treatment at the same time (Table 2). As shown in Fig. 3, rutoside was used as the pre-treatment as 4 h and 24 h before MB-PDT. The obtained results showed that, using rutoside 4 h prior MB-PDT can lead to a reduction in the cell viability compared to free MB under both dark and irradiation (PDT) conditions. Incubation of the A375 melanoma cells with rutoside for 24 h and then MB-PDT did not induce reductions in the cell viability, compared to free MB under the same condition. As indicated in Fig. 3c, adding rutoside for 24 before MB-PDT can lead to an increase in the cell viability under dark condition (dark toxicity of MB increased), and in irradiation it can lead to no reduction or increase in the cell viability. It could be suggested that, incubation for 24 h prior to MB-PDT caused the cell survival and rutoside has acted as antioxidant against the phototoxic effect of MB-PDT on cells.

### Post-treatment effect of rutoside on MB-PDT toxicity

**Fig. 4:**
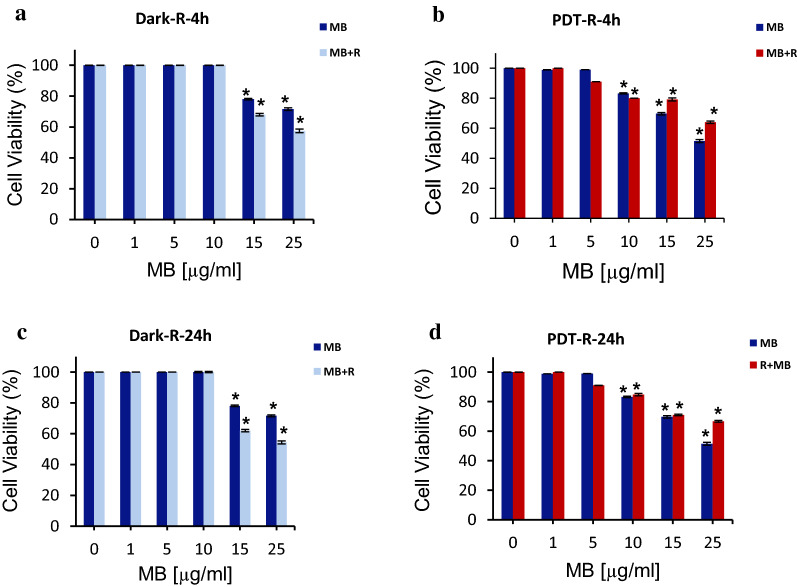
The cell viability of A375 melanoma cancer cells treated with various concentrations of MB and rutoside. MB treatment for 1 h and red irradiation (660 nm) for 90 s (PDT), then the treatment with rutoside (50 µg/mL) for 4 h (**a**, **b**) and 24 h (**c**, **d**). The results are expressed as mean ± SD (n = 3), *P < 0.05 (compared to the control (untreated) group. *R* rutoside

In another experiment, we used rutoside as post-treatment after treating the cells with MB-PDT. As presented in Fig. 4, treating the A375 melanoma cells with rutoside for 4 h and 24 h after the MB-PDT treatment, resulted in a slight reduction in the cell viability of the cells under dark condition, compared to MB free groups. In the case of irradiation (PDT), post-treatment with rutoside in both 4 h and 24 h caused an incraesed cell viability. It means that, under this condition (post treatment), rutoside increased the dark toxicity of MB; and on the other hand, it reduced the phototoxic effect of MB in the photodynamic treatment.

**Fig. 5:**
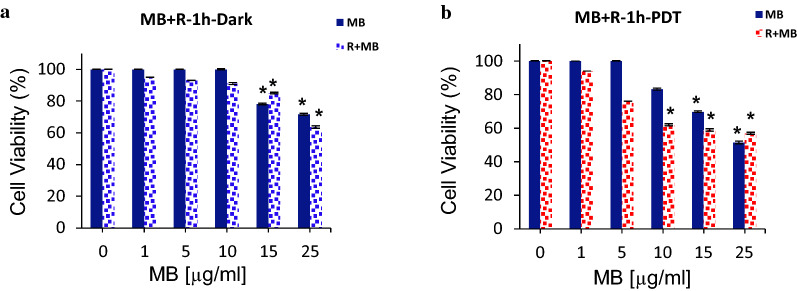
The cell viability of A375 melanoma cancer cells treated with various concentrations of MB and 50 µg/mL of rutoside. Rutoside(50 µg/mL) and MB treatment for 1 h, and then kept in dark (**a**) or red irradiation (660 nm) for 90 s (PDT) (**b**). Data are representative of three independent experiments and are expressed as mean ± SD (n = 3). *P < 0.05 (compared with control (untreated) group. *R* rutoside

Furthermore, another experiment was designed to investigate the effect of rutoside and MB-PDT simultaneously on the A375 cells. For this experiment, the cells were treated with rutoside and MB for 1 h, and then one group was kept in darkness and another irradiation with red light (PDT). As it can be observed in Fig. 5, this treatment led to a slight reduction in the cell viability of A375 cellsas compared to free MB group in both darkness and PDT group.

**Fig. 6:**
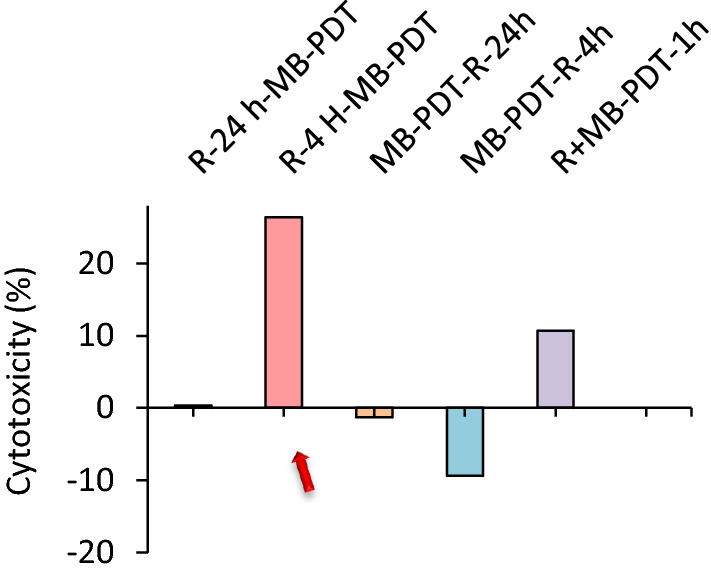
The cytotoxicity of rutoside (50 µg/mL) and MB-PDT on A375 melanoma cancer cells in different treatments as described in graph. *R* rutoside

From the obtained result, it can be suggested that, the rutoside has the optimum effect on the increasing phototoxic effect of MB-PDT on A375 melanoma cells when it was applied 4 h before MB-PDT (Fig. 6). For further experiments, we have considered this state and performed more experiments for understanding the mechanism of rutoside effect on MB-PDT.

### Effect of rutoside and then MB-PDT on the HDF normal cells

**Fig. 7:**
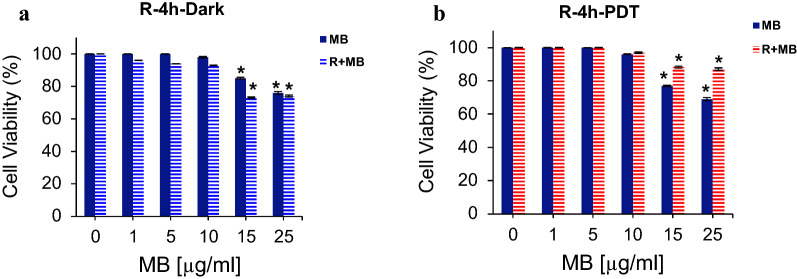
The cell viability of HDF cells treated with various concentrations of rutoside (50 µg/mL) for 4 h, and then MB treatment for 1 h and kept in dark (**a**), red irradiation (660 nm) for 90 s (PDT) (**b**). Data are representative of three independent experiments and are expressed as mean ± SD (n = 3). *P < 0.05 (compared with control (untreated) group. *R* rutoside

To be sure, this method has little toxic effects on normal cells, the human normal fibroblast cells, HDF cell lines, were treated firstly with rutoside and then MB-PDT. Our study showed that, the treatment of HDF cells with rutoside for 4 h and then MB-PDT can lead to increasing the cell viability of normal cells (reduction in dark toxicity of MB), and there was no significant reduction in phototoxic effect of MB-PDT (Fig. 7).

### Morphological assessments of cancer cells after rutoside and then MB-PDT

**Fig. 8:**
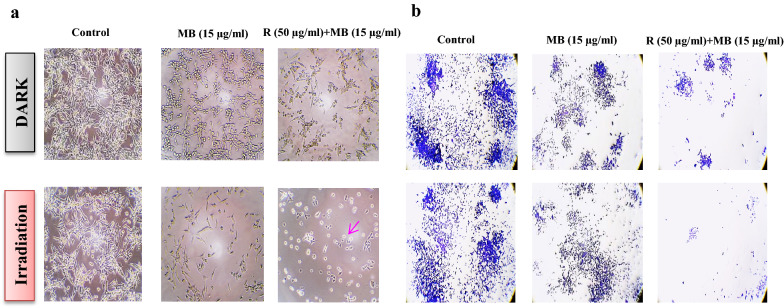
Invert microscopy images (×20) (**a**) and colony-forming ability (×20) (**b**) of A375 melanoma cancer cells after the treatment with rutoside for 4 h and then MB-PDT. *R* rutoside

To investigate the effect of rutoside treatment and then MB-PDT on the morphology of A375 melanoma cancer cells, the cells were treated with 50 µg/mL of rutoside and then with 15 µg/mL of MB for 1 h, which were later irradiated with 660 nm at 3 J/cm^2^ for 90 s (PDT). The cells were studied using invert light microscopy (20×). Figure 8a represents the A375 cells in 0 (control), 15 µg/mL of MB, and rutoside (50 µg/mL) + MB (15 µg/mL) under the dark and PDT conditions. As can be seen, by adding rutoside concentration at 50 μg/mL before MB-PDT, the number of cells remarkably decreased along with the morphology of the cells that changed from spindle to rounded shape and most of cells were dead. Figure 8b represents the colony-forming ability of A375 melanoma cancer cells in the presence of rutoside and then MB-PDT, as shown, the colonies were further decreased compared to free MB or control groups.

### Apoptosis detection in A375 melanoma cancer cells after the treatment with rutoside then MB-PDT

**Fig. 9:**
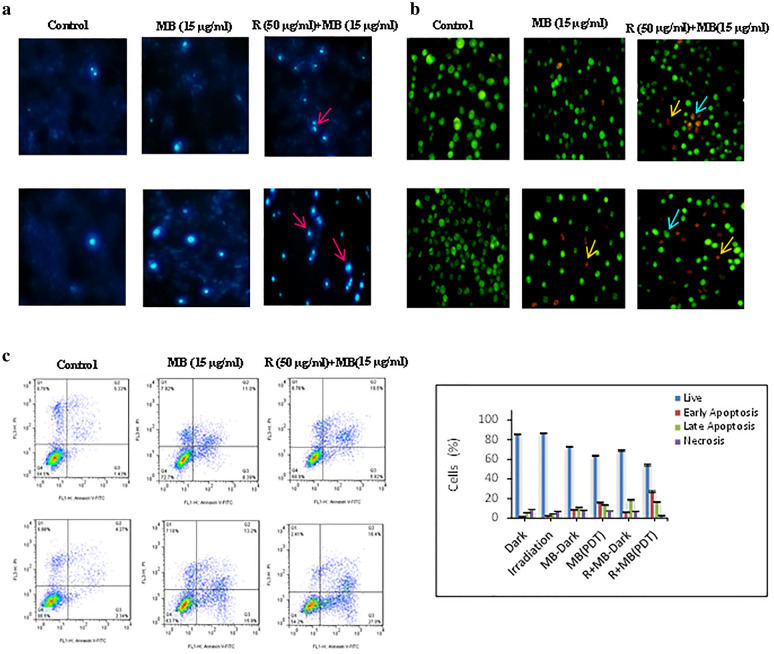
Flouresence microscopy and flow cytometry analysis of A375 melanoma cancer cells after the treatment with rutoside for 4 h and then MB (1 h)-PDT. Hoechst staining (**a**), AO/EB double staining (**b**), the apoptotic rates (annexin V-FITC/PI dual staining) (**c**). Histogram shows percentages of apoptotic and necrotic cells in the dark and irradiation groups (**d**). *R* rutoside. Data are representative of three independent experiments and are expressed as mean ± SD (n = 3)

Figure 9 shows the morphological changes of A375 cells with AO/EB dual and hochest staining using fluorescence microscopy. Using Hoechst staining, control cells showed very faintly and homogeneous staining of their nuclei, but rutoside treatment and then MB-PDT treated cells showed a strong blue fluorescence (Fig. 9a). As it can be seen in the control group (0 μg/mL of rutoside and MB), the cells that represent the shape of live cells are indicated by green color. By adding rutoside at 50 μg/mL, before MB-PDT, the nuclei of cells changed to orange-red cells showing the early/late apoptosis. Under irradiation and in the pre-treatment with of rutoside, the melanoma cells showed the characteristic of apoptotic cells with chromatin condensation and nuclear fragmentation. It suggests that, in the pre-treatment with rutoside and then MB-PDT, the cells intend to death (Fig. 9b).

For insight into the death mechanism of each condition, the flow cytometry assay with annexin/PI was performed. As can be seen in Fig. 9c, by adding the rutoside before MB treatment and also in the presence of irradiation, the number of apoptotic cells in early or late stages more increased in comparison to free MB or control groups.

### Effect of rutoside and MB-PDT on ROS generation in melanoma cancer cells

**Fig. 10:**
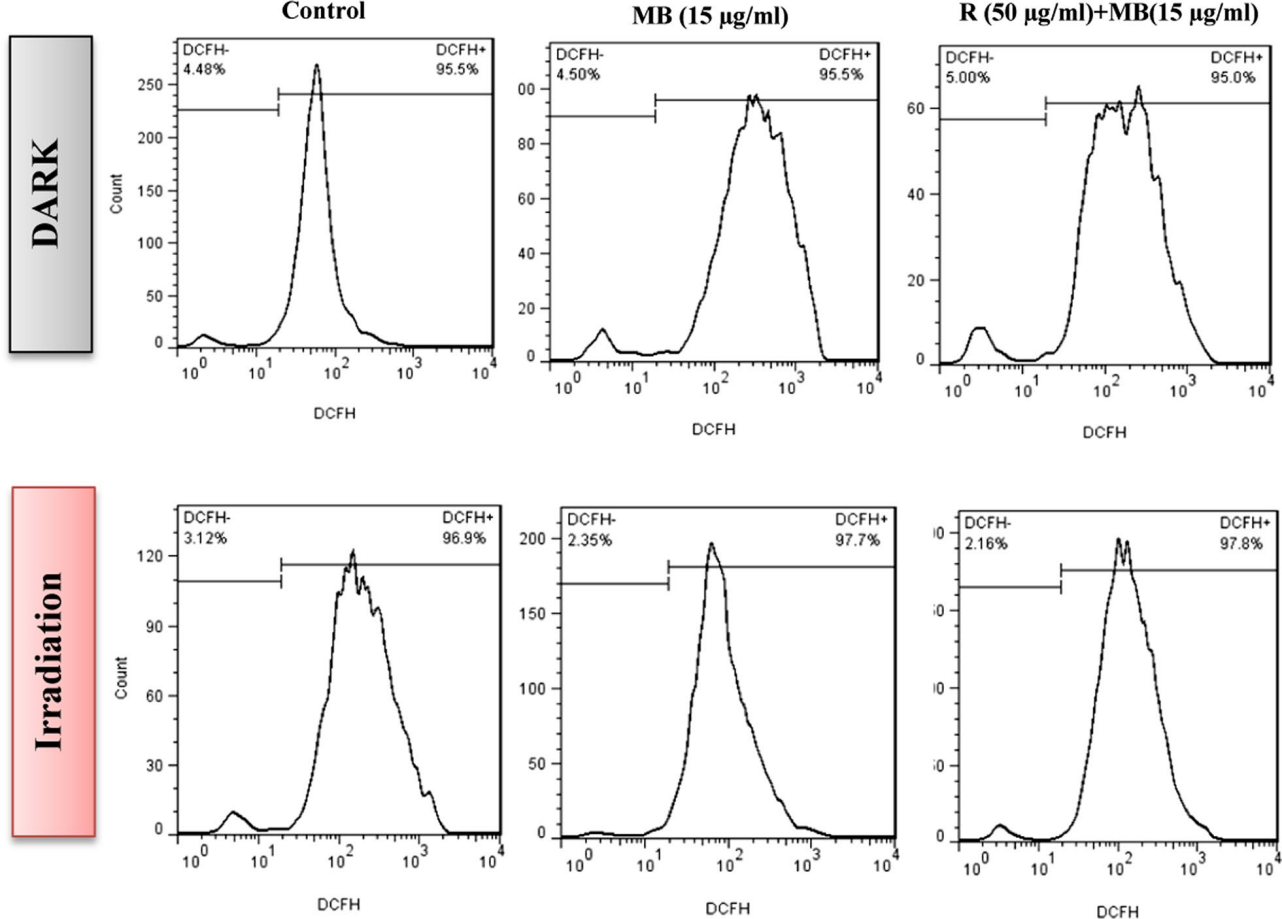
Effects of rutoside (50 μg/mL) as pre-treatment and then MB-PDT on intracellular ROS generation in A375 cells. The cells were stained with DCFH-DA (2 mM), and analyzed by flow cytometry. *R* rutoside

As presented in Fig. 10, the ROS production after the treatment with rutoside and then MB-PDT increased in A375 melanoma cancer cells compared to the free MB and control groups. It can be suggested that, ROS production could act as one of the main factors in the death mechanism of cancer cells using rutoside as pre-treatment and then MB-PDT.

### Cell cycle alteration after rutoside treatment then MB-PDT

**Fig. 11:**
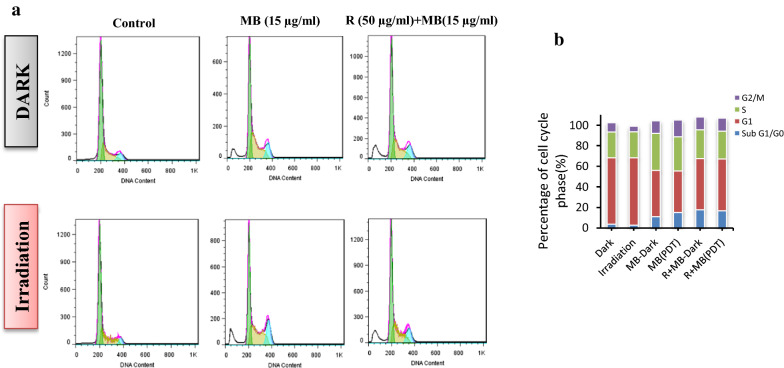
Flow cytometry histograms for performing the cell cycle analysis of A375 cells untreated (Control) or treated with 50 μg/mL rutoside and then MB (15 μg/mL) for 1 h followed by red laser irradiation (PDT). The treated cells were stained with PI and were then analyzed by flow cytometry (**a**). X-axis indicates DNA content, and the Y-axis displays the cell count. Plots depict the variation of the percentage of cells in each phase of the cell cycle (**b**). The data are shown as the mean ± standard deviation of three experiments. *P < 0.05 represent the significant difference vs the control

As shown in Fig. 11a, rutoside treatment and then MB-PDT induced a significant G0/G1 phase arrest in A375 melanoma cancer cell. When the A375 cells were incubated with 50 μg/mL rutoside and then MB (15 μg/mL) for 1 h followed by red laser irradiation (PDT), an accumulation of cell population in a G0/G1 phase increased from 2.73% in the control group (only irradiation) up to 16.8% accompanied by a decrease in the percentage of the S and G2/M phases of the cells treated with free MB-PDT (Fig. 11b).

## Discussion

Rutoside, also known as quercetin-3-rutosideoside, and sophorin; is a flavonol glycoside compound mainly found in buckwheat [46, 47]. Rutoside has been the subject of special anti-cancer effects [40]. Research suggested that, rutoside can be a useful adjunct to radiotherapy [35]. It has more advantageous over other flavonoids, as it is a nontoxic and also a nonoxidizable molecule [39]. Photodynmaic treatment (PDT) is a non-invasive method relying on three important ingredients including: light, oxygen, and a photosensitive compound known as photosensitizer (PS) [48]. The PS molecules after entering the cell, absorbing the light at relevant wavelength, and beginning processes result in the selective damage of the unwanted cells [49].

**Fig. 12:**
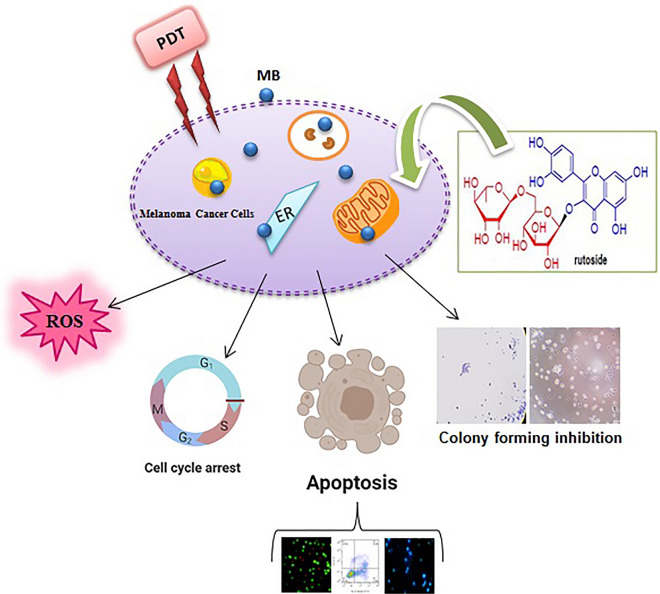
Shematic illustarion of changes happened in A375 melanoma cells after treating with rutosid and MB-PDT

According to the obtained Gibbs free energies for each form of monomeric and dimeric MB, it is clear that the interaction of MB with rutoside is a molecular interaction. By considering red shift in maximum peaks of MB spectra (monomeric and dimeric forms of molecule) by increasing rutoside concentration, it can be concluded that, the hydrophobicity of chromophore microenvironment increases. Also, the Gibss free energy for monomeric MB interaction with rutoside is lower (− 19.28 kJ/mol) than the dimeric MB (− 14.03 kJ/mol). It means that, monomeric MB has higher potential for interaction with rutoside in comparison with dimeric form. MB and rutoside make molecular interactions by major hydrophobic forces, as running interaction forces. Accordingly, it seems that mixed micelle complex is produced due to the above-mentioned interactions (increasing hydrophobicity of chromophore microenvironment). Moreover, these interactions can cause dissociation of the dimeric MB from monomeric form due to the obtained Gibss free energies for interactions. To the best of our knowledge, the monomeric MB is the best active form of MB for photodynamic activities. Also, it is clear that MB is a redox active molecule that can be oxidized with oxidative compound in around. Therefore, micellization by rutoside molecules can protect the MB as photosensitizer for better photodynamic activity. These effects can increase the photodynamic activity of MB in cancer therapy. For proving these effects, we consider to examined the photodynamic activity of MB in the presence of rutoside on cancer cells. In the present study, we demonstrated that, rutoside and MB-PDT suppressed the cells proliferation by inducing G0/G01cell cycle arrest and promoting apoptosis in A375 cells. As it is known, the main biological characteristics of tumor cells are uncontrolled proliferation and higher migration and colonization abilities. The MTT assay results showed that, rutoside plus MB-PDT significantly suppressed the A375 cell viability and also proliferation in a dose-dependent manner. The cell cycle is the number of events that happen in a cell lead to cell division and duplication (replication); also regulation of the cell cycle is crucial for the survival of a cell. The studies showed that, PDT and also flavonoids can promote cell cycle arrest in individual phases that is a major anticancer effect [50]. Our results stated that, rutoside plus MB-PDT induced G0/G1 phase arrest in A375 cells. Cell cycle regulation is also essential in interceding radiosensitivity. Cells are mostly sensitive to radiation during the G2/M phase, less sensitive during G1, and least sensitive near to the end of the S phase [51]. Our results showed that rutoside pre-treatment made the A375 cells sensitive to G1 arrest during PDT. As it is well-known, apoptosis is the main reason of cell death induced by antitumor drugs. Here, we indicated that, using rutoside as pre-treatment before MB-PDT could increase the cells in early and late apoptosis phases compared to control as confirmed by fluorescence microscopy. In our study, we found that, pre-treatment with rutoside could increase the ROS generation that was induced by MB-PDT(Fig. 12).

## Conclusion

Various preclinical studies and number of clinical trials suggested that, the use of PDT in combination with other treatments may be of benefit as compared to the individual modalities. The result show that, rutoside and MB-PDT have cytotoxic and antiprolifrative effects on A375 human melanoma cancer cells, while their effects on human normal cell were not significant. MB-PDT and rutoside combination induced apoptosis and cell cycle arrest in human melanoma cancer cell line. Intracellular ROS increased in A375 cancer cell lines after the treatment with rutoside and MB-PDT. The results suggested that rutoside and MB-PDT could be considered as novel approaches in the combination with photodynamic treatment. However, lots of questions regarding signaling pathways still remained unanswered and more work is required to elucidate the exact mechanisms. We recommended performing further investigation of this combination with outlining its potential against melanoma cancer in in vivo studies.

## Data Availability

The datasets generated and analyzed during the current study are available from the corresponding authors on reasonable request by permission of institute and department chairman’s.
